# Whole-genome detection using multivalent DNA-coated colloids

**DOI:** 10.1073/pnas.2305995120

**Published:** 2023-09-05

**Authors:** Peicheng Xu, Ting Cao, Qihui Fan, Xiaochen Wang, Fangfu Ye, Erika Eiser

**Affiliations:** ^a^Beijing National Laboratory for Condensed Matter Physics and Laboratory of Soft Matter Physics, Institute of Physics, Chinese Academy of Sciences, Beijing 100190, China; ^b^Wenzhou Institute, University of Chinese Academy of Sciences, Wenzhou, Zhejiang 325001, China; ^c^School of Physical Sciences, University of Chinese Academy of Sciences, Beijing 100049, China; ^d^Porelab, Department of Physics, Norwegian University of Science and Technology, Trondheim NO-7491, Norway; ^e^Cavendish Laboratory, University of Cambridge, Cambridge CB3 0HE, United Kingdom

**Keywords:** full genome detection, superselectivity, multivalency, DNA detection, colloidal probes

## Abstract

To minimize the incorrect use of antibiotics, there is a great need for rapid and inexpensive tests to identify the pathogens that cause an infection. The gold standard of pathogen identification is based on the recognition of DNA sequences that are unique for a given pathogen. Here, we propose and test a strategy to develop simple, fast, and highly sensitive biosensors that make use of multivalency. Our approach uses DNA-functionalized polystyrene colloids that distinguish pathogens on the basis of the frequency of selected short DNA sequences in their genome. Importantly, our method uses entire genomes and does not require nucleic acid amplification. Polystyrene colloids grafted with specially designed surface DNA probes can bind cooperatively to frequently repeated sequences along the entire genome of the target bacteria, resulting in the formation of large and easily detectable colloidal aggregates. Our detection strategy allows “mix and read” detection of the target analyte; it is robust and highly sensitive over a wide concentration range covering, in the case of our test target genome *Escherichia coli* bl21-de3, 10 orders of magnitude from $10^{1}$ to $10^{10}$ copies/mL. The sensitivity compares well with state-of-the-art sensing techniques and has excellent specificity against nontarget bacteria. When applied to real samples, the proposed technique shows an excellent recovery rate. Our detection strategy opens the way to developing a robust platform for pathogen detection in the fields of food safety, disease control, and environmental monitoring.

## Significance

There is a great need for easy tests identifying infection-causing bacteria. Identifying bacterial DNA using the polymerase chain reaction may be problematic in low-tech environments. Here we present and test a method to identify whole bacterial genomes without using DNA amplification. Our experiments validate an approach proposed in simulations (PNAS 117, 8719–8726 (2020)). We exploit the super-selectivity of the binding of frequently repeating, short nucleotide sequences in the bacterial genome to complementary ssDNA grafted on polystyrene colloids. Using this approach, we observed that solutions with as few as 5 copies/mL of *E.coli* bl21-de3 genome, resulted in a strong and selective cluster-growth of the colloids. Our approach is generic and could greatly facilitate early pathogen detection.

In a world at constant risk from pandemics, there is a great need for cheap, fast, and robust techniques to identify the pathogens that cause a particular disease, thereby helping to select the correct intervention. The lack of suitable pathogen identification tools is dangerous: For instance, antibiotics, which target bacteria, are often prescribed inappropriately for viral infections.

There are, of course, many pathogen tests available, and among them, the one based on the PCR is considered the best in terms of sensitivity and selectivity. However, PCR tests need to be processed in a lab, which limits their applicability in environments where the necessary facilities are not available, Moreover, as a pathogen mutates, one or more of the original DNA sequences that the PCR targets may change.

Traditionally, bacterial pathogen diagnosis was based on growing pathogens in the lab. For the pathogens that can be cultured, the approach is “low-tech,” but it requires a long diagnosis cycle (typically days), and is susceptible to contamination (1, 2). Real-time and semiquantitative detection can be achieved with immunology-based technologies like enzyme-linked immunosorbent assay (ELISA) and lateral flow assay (LFA), which are based on specific antigen–antibody reactions. The applicability of LFA is constrained by the cost of antibody synthesis, their (often) limited sensitivity, and their susceptibility to contamination (2, 3). Nucleic acid–based approaches, including various types of PCR, involve the amplification and hybridization of selected DNA/RNA sequences of target pathogens. PCR methods tend to be the gold standard of pathogen identification, as they combine high sensitivity and specificity with a reasonable detection speed. However, PCR requires relatively expensive reagents and, more importantly, sophisticated laboratory equipment combined with the technical expertise to run the tests (1, 4). There are, of course, many other biosensing techniques, e.g., those based on electrochemical platforms. However, these powerful techniques also require advanced equipment and technical expertise (5, 6).

The above discussion illustrates the continued need for simple, fast, cheap, and robust diagnostic methods that combine a high selectivity with a high sensitivity. Basically, what we need is a technique that combines the power of DNA-based techniques with the simplicity of, say, a lateral flow test.

**Fig. 1.**
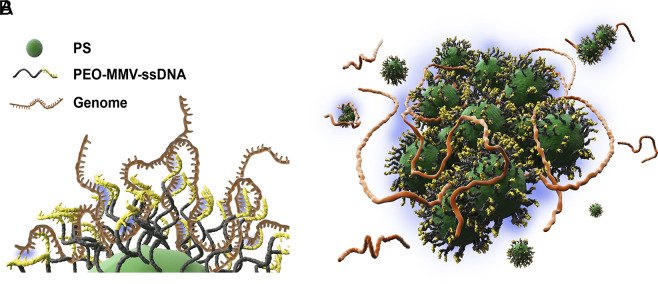
(*A*) Cartoon of a DNA-coated colloidal particle bound to parts of a long bacterial genomic DNA. Short single-stranded DNA probes, which are grafted on the surface of a colloid, form complementary connections with the target genomic DNA. (*B*) Colloidal aggregation and subsequent sedimentation resulted from the cooperative binding of many DNA-coated particles to denatured bacterial DNA.

Here, we report the design and testing of a sensitive and selective diagnostic system based on the concept of whole-genome detection, following the strategy proposed in the theoretical analysis by Curk et al. (7). In our approach, we use micron-scale polystyrene (PS) particles, grafted with 20-nucleotide (20-nt) long single-stranded DNA (ssDNA) fragments designed to hybridize multivalently with the most frequent complementary sequences on genomic ssDNA of the target (in our example: *Escherichia coli* bl21-de3) – This is illustrated in Fig. 1. Here, and in what follows, we use the term multivalency to denote the situation where a probe molecule or particle is coated with several (often many) chemical units (“ligands”) that can bind weakly and reversibly to complementary units (“receptors”) on the target surface. A single ligand may interact only slightly more strongly with a cognate than with a noncognate receptor. However, many multivalent contacts are needed for binding, thereby amplifying the binding free-energy difference of the multivalent probe to surfaces coated with cognate and noncognate receptors. Hence, multivalency improves selectivity. Often, the ligands on a multivalent probe can bind in many different ways to the same set of receptors. Consider, for instance, a multivalent, linear chain molecule: If the receptors are sufficiently close together, many permutations of the ligands, corresponding to many conformations of the chain molecule, can bind to the same receptors. The number of such permutations may be very large: The logarithm of this number determines an entropic gain on binding. This additional contribution to the probe-substrate-binding free energy is strongest for surfaces with a higher receptor concentration. The high sensitivity of the binding strength of multivalent probes to the receptor density is called “superselectivity.” For a discussion of the more general situation, see ref. 8.

The probe ssDNA sequences on the colloidal surfaces were selected using the algorithm of ref. 7, which identifies the best targets for multivalent binding. In what follows, we refer to the maximally multivalent probe sequence that we use as the MMV probe. For the sake of comparison, we also tested two probe sequences that should not benefit from multivalency, namely one that is a 20-nt poly-A strand (referred to as PolyA) and one probe of the same length that contained a random sequence of nucleotides (referred to as RAN).

The *E. coli* genome that we considered in this study is around $10^{6}$ base pairs long (9) and can bind many colloids simultaneously, which facilitates visual detection of the genome-induced colloidal clusters: In the presence of the target genome, the colloids will cluster and sediment, which can be easily monitored using a microscope.

The use of short ssDNA probes has several advantages: They tend to be biocompatible and exhibit a strong binding affinity and a high target selectivity. The 20-nt probes that we use tend to be relatively inexpensive and are easy to vary. Moreover, they can be functionalized to form chemical bonds with complementary groups on the colloid surface. Because of their short length, the ssDNA probes can be packed densely on the colloidal surface (4, 10–12).

Of course, nanoparticles have been used extensively to immobilize bioreceptors (3, 10, 13, 14), and DNA–nanoparticle conjugates have been used extensively for the detection of viral and bacterial strains (15, 16). However, to the best of our knowledge, the action of the existing techniques is based on monovalent binding between surface DNA probes and a particular segment of the target genome, which means that the pathogen DNA has to be amplified before it can be detected.

As we show below, our sensing system can operate at a “mix and read” detection speed; it has good diagnostic sensitivity with a wide dynamic detection range from $10^{1}$ to $10^{10}$ copies/mL as well as a limit of detection down to ≈ 5 copies/mL without the need of PCR amplification. Importantly, we demonstrate that the multivalent approach can discriminate against 6 nontarget bacteria and has a good recovery rate in real samples.

The theory behind multivalent binding to target surfaces with a high “receptor” density was developed in a paper by Martinez and Frenkel (17). This paper showed how multivalent probes can be extremely sensitive (superselective) to the concentration of the target receptors on a surface. This phenomenon was subsequently demonstrated in experiments on surfaces coated with nonbiological ligand–receptor pairs (see, e.g., refs. 18 and 19).

## Experimental Section

### Materials.

Polydimethylsiloxane (PDMS), tetrahydrofuran (THF), x10 phosphate-buffered saline (PBS), 4’,6-diamidino-2-phenyl-indole, dihydrochloride (DAPI), x10 Tris–EDTA (TE), ascorbic acid, and sodium chloride (NaCl) were purchased from Beijing BioDee Biotechnology. Dragon Green fluorescence polystyrene spherical colloids (diameter: 0.513 μm) were obtained from Bangs Laboratories Inc., and ω–azide end-functionalized polystyrene-b-poly (ethylene oxide) diblock copolymer (PS = 1600 gmol${}^{-1}$, PEO = 5000 gmol${}^{-1}$) from Polymer Source. Alkyne-modified and fluorescent, cy5-modified single-stranded DNA (ssDNA) were both custom-synthesized by Sangon Biotech. The genomic DNA of *Escherichia coli* bl21-de3 (*E. coli*), *Pseudomonas aeruginosa* (*P. aeruginosa*), *Staphylococcus aureus* (*S. aureus*), *Bacillus subtilis* (*B. subtilis*), *Acinetobacter baumannii* (*A. baumannii*), *Shigella*, and *Listeria monocytogenes* (*L. monocytogenes*) were provided by Beijing Zoman Biotechnology Co., Ltd., and Mingzhou Biotechnology Co., Ltd. All reagents were of analytical grade and used without further purification.

### Functionalizing PS Colloids with Reactive PS-b-PEO-N${}_{3}$ Groups.

The grafting of PS-b-PEO-N${}_{3}$ onto PS particles was carried out using a modified protocol (20, 21) described by Yi et al. (22). In this procedure, 150 μL of PS suspension (10 mg/mL) was combined with 2,000 μL of PS-b-PEO-N${}_{3}$ aqueous solution (2.5 mM). In order to swell the PS colloids so that the PS block of the diblock copolymer can be incorporated into the surface of the colloids, the mixture was poured into 2,200 μL THF to bring its volume fraction to over 50%. The mixture was shaken thoroughly at room temperature for 2 h. Next, for the purpose of deswelling the PS particles, an excess of deionized (DI) water was added to the hybrid suspension to bring the volume fraction of THF below 10%. The sample was evaporated in a fume hood for 2 h to remove the THF. When finished, the suspension of colloidal particles (referred to as N${}_{3}$-PS) was purified at least 3 times by washing it with DI water and then stored at 4 ${}^{{}^{\circ}}$C for further use.

The immobilized azide-functionalized PEO polymers form a dense brush on the colloidal surface and stabilize them against spontaneous (i.e., not genome-induced) aggregation.

### DNA Grafting onto N${}_{3}$-PS Using Click Chemistry.

200 μL of PBS buffer (x10), 110 μL of (100 μM) alkyne-modified maximally multivalent ssDNA probe (MMV: alkyne-5’-CGGATGCGGCGTGAACGCCT), 30 μL of N${}_{3}$-PS suspension, 20 μL of ascorbic acid (1 M), and 40 μL of CuSO${}_{4}$ aqueous solution (0.1 M) were added sequentially to 1,600 μL of DI water to reach a total volume of 2 mL. The grafting of target-specific DNA probes onto N${}_{3}$-PS particles was realized through a strain-promoted azide–alkyne cycloaddition reaction, which was carried out by shaking the hybrid solution vigorously overnight at ambient conditions. Finally, DNA-functionalized PS particles (coded as MMV-PS) were purified by centrifugation, resuspension in deionized water, and stored at (4 ${}^{{}^{\circ}}$C). In order to demonstrate the success of DNA attachment, cy5-modified ssDNA (cy5-5’-AGGCGTTCACGCCGCATCCG), which is fully complementary to the surface DNA probe, was mixed with the MMV-PS suspension and then observed under a confocal fluorescence microscope. Strong red fluorescence was only observed for PS particles with MMV functionalization (*SI Appendix*, Fig. S2), which is indicative of the successful grafting of surface DNA probes. SEM images, shown in *SI Appendix*, Fig. S1, confirm that the incorporation of the block copolymer and probe DNA did not result in noticeable changes in particle size, shape, or surface morphology. We note that the preparation of the DNA-coated PS colloids is straightforward and scalable. Moreover, they can be stored at room temperature for future use: Our multivalent detection system maintained its performance over the course of a month (*SI Appendix*, Fig. S8), which should be a bonus for practical applications.

### Detection of the Bacterial Genome by DNA-Decorated PS Particles.

The denaturation of the bacterial genome to create genomic ssDNA was achieved by heating genomic samples at 95 ${}^{{}^{\circ}}$C for 10 min in a Thermal Cycler (Bio-Rad®, C1000 Touch, U.S.A.). Subsequently, samples could either be used immediately or be refrigerated to −20 ${}^{{}^{\circ}}$C for future use.

50 μL of MMV-PS was added to 50 μL of genomic DNA solution (g/L) followed by the addition of 10 μL of TE buffer containing 6 mM NaCl. 5 μL of DAPI was added to the mixtures, and the whole sample was incubated for 10 min with mild shaking. Following that, 20 μL of the mixture was loaded slowly into a poly-dimethylsiloxane (PDMS) chamber (a hollow PDMS cylinder sandwiched by two glass slides.) Both the PDMS and the glass were washed with 75% ethanol and pretreated with plasma to prepare them for use in a confocal fluorescence microscope.

### Human Test Samples.

We tested our biosensing system using spiked healthy human urine. First, human urine was collected from a healthy person (age 30). Next, *E. coli* bl21-de3 was cultured in the human urine solution, and then, the genomic DNA was extracted. The final urine sample contained $∼10^{5}$ copies/mL. After denaturation, the sample was then tested using the established mix of MMV colloidal probes and DAPI fluorophore. All experiments were undertaken at least 3 times.

### Characterization.

The incorporation of diblock copolymer into the PS particles was verified using a cold-field emission scanning electron microscope (SEM) (Hitachi®, Regulus 8230, Japan). The concentration of genomic DNA was determined with a spectrophotometer (Thermo Fisher Scientific®, Nanodrop2000C, U.S.A.). Fluorescence microscopy images of surface DNA attachment and the cooperative binding between MMV-PS particles and bacteria genomic DNA were taken using a confocal microscope (Leica TCS SP8®, Germany). In *SI Appendix*, we describe how we estimated the optimal operating conditions for full-genome detection with multivalent DNA-coated colloids.

## 1. Results and Discussion

### Proof of Concept.

As sketched in Fig. 1, the working principle of the proposed biosensor is the target genome–induced aggregation of the DNA-functionalized PS particles. The formation of colloidal aggregates is detected optically.

As a first test, we used our DNA-colloid system to detect bacteria of the *E.coli* family. *E. coli* is one of the most prevalent pathogenic bacteria. It is responsible for a wide range of medical conditions (23–25). In the absence of the infrastructure needed to perform PCR, it may take 2 to 3 d to diagnose this pathogen.

Here, we chose the nonpathogenic *E. coli* bl21-de3 as a proof-of-concept target. The length and sequence of the target-specific DNA probes (MMVs) were designed and optimized by several rounds of experiments and computer simulation, enabling a single DNA probe to bind cooperatively to multiple sites throughout the target genome (7). As the *E. coli* bl21-de3 genome comprises O($10^{6}$) base pairs, a single genome can bind to a large number of PS particles coded with this MMV probe sequence. In the present case, the 20-nucleotide (nt) MMV probe can bind to 79 complementary sequences along the genome. We note that these copies are almost equally distributed among the two complementary strands of the denatured, but still knotted, bacterial plectoneme. As argued in ref. 7, the actual multivalency is higher than 79, as the 20-nt MMV contains substrands with a higher repeat frequency. The key point to note is that a judicious choice of the target repeat sequence makes it possible to distinguish even between quite similar pathogenic or nonpathogenic microorganisms. This selectivity is important as many clinical samples (especially urine) may contain harmless symbiotic bacteria/viruses, in addition to pathogens that cause infections.

In spite of its selectivity, our detection strategy is robust with respect to small numbers of point mutations: Disabling one or two of the target sequences out of several dozens will barely affect the ability of a genome to induce aggregation.

In a first test, we denatured the genomic target analyte *E. coli* bl21-de3 in a Thermal Cycler. Rapid cooling on ice prevents recombination of the complementary genomic ssDNAs and can be used immediately (26). The suspension of MMV-functionalized PS particles was then added to our denatured *E. coli* solution and mixed, allowing the ssDNA strand on the colloids to hybridize with the short, complementary sequences in the genome (Fig. 1*A*).

**Fig. 2.**
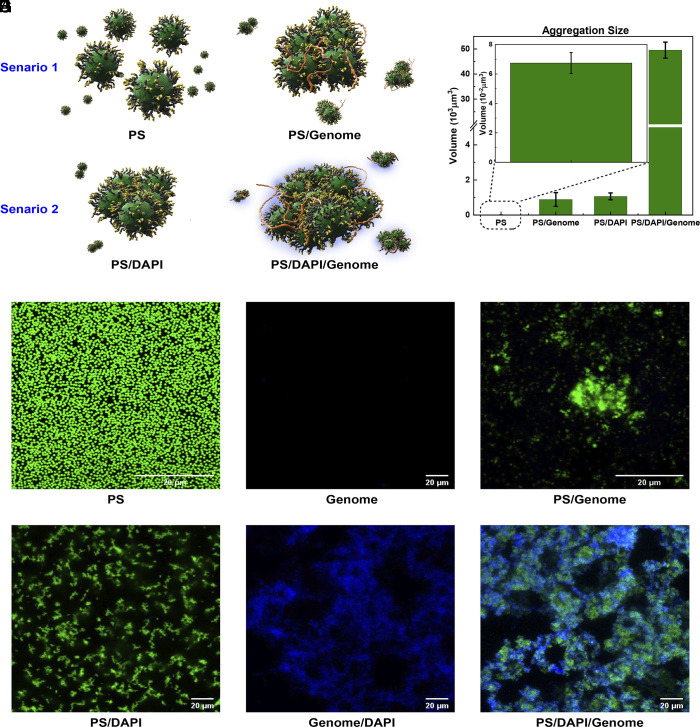
Aggregation behavior of MMV-functionalized PS particles and the denatured *E. coli* bl21-de3 genome. (*A*) Illustration of the effect of the double-stranded DNA stainer DAPI. *Top Left*: suspensions of MMV-PS particles show no aggregation. *Top Right*: Upon addition of single-stranded genome, relatively small aggregates are formed. *Bottom Left*: The addition of the double-stranded DNA stainer DAPI causes weak aggregation of the MMV-PS particles on their own. *Bottom Right*: Adding both DAPI and target genome causes the formation of large aggregates. (*B*) Average aggregation sizes of the MMV-PS-particle clusters observed for these four conditions in (*A*), based on confocal images taken in the green fluorescence mode. Images (*C*–*H*) show confocal microscopy snapshots of the different systems studied. Every image corresponds to a slice of thickness Δz= 1.48 μm. (*C*): green fluorescent PS colloids distributed uniformly throughout the sample. (*D*): target genome only (no fluorescent staining). (*E*): Fluorescently labeled MMV-PS particles mixed with the target genome (no DAPI). Confocal images (*F*–*H*) show the effect of the blue-fluorescent dye DAPI, which binds to dsDNA: (*F*): MMV-PS colloidal suspension mixed with DAPI only. (*G*): DAPI-stained nondenatured (double-stranded) genome. (*H*): MMV-PS, DAPI, and target genome mixture showing strong, colocalized aggregation.

We performed confocal microscope (CM) measurements to quantify the degree of DNA-induced colloidal aggregation by measuring the size of the colloidal aggregates formed. We stress that the presence of genomic material affects the size of the aggregate, rather than the green fluorescent intensity, as even isolated colloids will fluoresce. Further, we tested whether the genomic ssDNA had hybridized (bound) to the DNA probes on the colloid surfaces. Hybridization was detected by using the dye DAPI, which exhibits blue fluorescence when it binds to the minor groove of double-stranded DNA (Fig. 2 *G* and *H*). We found that the size of the aggregates that formed under the influence of genomic DNA was such that detection by a low-resolution microscope would have sufficed. CM images taken in control experiments were also performed to verify that the denatured *E. coli* solutions did not bind to either bare PS beads or to those functionalized with the N${}_{3}$-block copolymer brush (*SI Appendix*, Fig. S3*C*). The results shown in the following were all performed using solvent and probe conditions that were optimized with respect to the PS-b-PEO-N${}_{3}$ diblock copolymer, which acted as an anchor for the MMV strands, the number of MMV strands attached to the polymer brush, the effect of the Cu${}^{2+}$ concentration needed to perform the covalent binding of the MMV strands to the polymer brush, and the effect of the salt (NaCl) concentration in the final sample, which is necessary for successful hybridization of the MMV strands to the genome. Details for this optimization are given in (*SI Appendix*, Figs. S3–S7). All following results were performed using these optimized colloidal probes.

The sketch in Fig. 2*A* illustrates how we tested that large aggregates were only formed in the presence of genomic ssDNA and that the presence of DAPI enhanced the size of the aggregates.

In the absence of both DAPI and target genome, the MMV-PS particles (denoted PS) do not aggregate but remain uniformly distributed (Fig. 2*C*). In the absence of MMV-PS particles, samples containing pure genome show no green fluorescence (Fig. 2*D*). Samples containing both PS colloids and the genome of *E. coli* bl21-de3, but no DAPI, show a few medium-sized green fluorescent aggregates (Fig. 2*E*): These aggregates had an average size of order ten colloidal diameters. When mixing MMV-PS particles with DAPI (scenario 2), no blue fluorescence was observed, which is to be expected as DAPI is a double-stranded DNA stain. However, the fluorophore promotes weak aggregation between the MMV-PS particles (*SI Appendix*, Fig. S2). Finally, when MMV-PS, DAPI, and genomic DNA were all mixed together, very large aggregates formed exhibiting both green and blue fluorescence, as shown in the overlay of the two separate fluorescent channels of CM (Fig. 2*H*). The large increase in aggregate size upon addition of both the target genome and DAPI illustrates the strong synergistic interaction between these two components. The green fluorescence stemmed from the PS particles in the aggregate, while the blue fluorescence is a sign of hybridization between surface DNA probes and the long bacterial genome.

The fact that the colloids form small clusters even in the absence of the target genome is probably due to the fact that DAPI can act as a bridge between ssDNA-coated surfaces. Evidence supporting the hypothesis that this DAPI-mediated bridging mechanism is caused by the presence of the ssDNA brush on the probe colloids comes from our tests in which we mixed the plain, green fluorescent PS colloids with either DAPI or DAPI and the genome (*SI Appendix*, Fig. S2). In both cases, no significant aggregation or blue fluorescence was observed. Similarly, no aggregation was observed when using colloids densely grafted with the N${}_{3}$-functionalized block copolymers (*SI Appendix*, Fig. S3). This suggests that the DAPI and the target genome act synergistically: The rapidly diffusing DAPI can first make small clusters of a few PS particles. But these colloidal clusters are then much better probes for the multivalent target genome. As a consequence, we typically observe the largest colloidal clusters in systems with the target genome and DAPI. In other words: DAPI is both a hybridization probe and an amplifier of the colloidal aggregation. We stress that this signal amplification did not cause any false positives because DAPI was only effective as an amplifier if the colloids were mixed with the target genomic DNA. Replacement of DAPI by Hoechst 33258, another blue fluorescent, intercalating dye for dsDNA did not show such pronounced aggregation effects.

The performance of the proposed biosensor was estimated by measuring the size of the colloidal aggregates from z-stacks of confocal images measured in the xy plane. It was sufficient to consider only the green fluorescent images showing the colloids. The volume of the aggregates was obtained by recording the area S measured in a xy-image at a given height z. The images were separated by a unit thickness (Δz= 1.48 μm) for each stack we recorded. For every layer n, we measured the cross-section $S_{n}$ of the fluorescent cluster in that layer. We then added the fluorescent cross-sections of all layers, and multiplied with the thickness Δz of the layer, to obtain the volume of the aggregate. For instance, for the sample containing only DNA-functionalized colloids, the area of one single fluorescent dot was 0.2 μm${}^{2}$, which was approximately equal to the cross-section area of a single PS particle. When mixing colloids with genomic DNA without the addition of DAPI, the aggregation size increased by a factor $10^{4}$ (Fig. 2*B*). With the assistance of DAPI, the aggregation area increased dramatically by another factor of fifty, reaching an aggregation volume of almost $10^{5}$μm${}^{3}$.

Next, we tested the binding selectivity of different kinds of surface DNA probes when targeting the same genome. Following the procedure described in the *Experimental*, we prepared PS colloids grafted with two designed ssDNA probes that did not correspond to a frequent repeat in the target ssDNA and which are therefore not expected to benefit from the advantage multivalency.

**Fig. 3.**
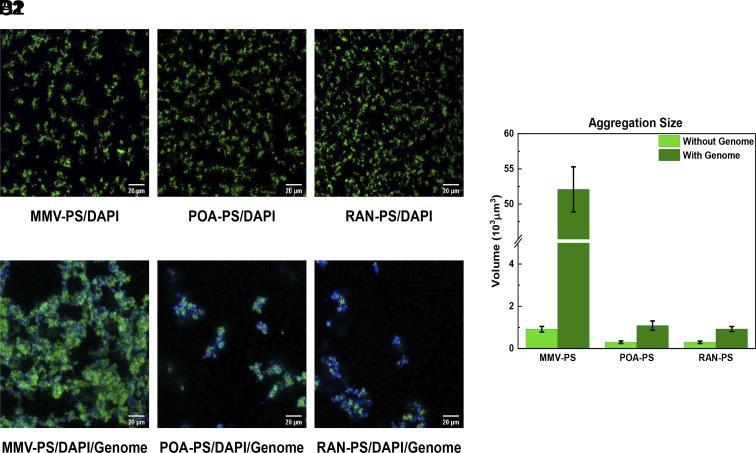
The selectivity of different surface DNA probes. (*A1*–*C1*) Confocal images of solutions containing PS particles functionalized with MMV, POA, and RAN probes all form small green aggregates when stained with DAPI. (*A2*–*C2*) When mixed with the target genome, only MMV-functionalized PS colloids form big aggregates, while both POA and RAN-coated PS probes only form small clusters. (*D*) The comparison of aggregation size for the different DNA probes, extracted from the green fluorescent confocal images.

These probes served as baseline comparisons with the MMV probe: PolyA was a 20-nt poly “A” sequence while RAN (GGAGCTCGGAGGTGATGGG) stood for a random sequence, generated using a quasi-random number generator. The differently functionalized PS particles all formed small patches (Fig. 3 *A1*–*C1*) of similar size as when stained with DAPI alone, indicative, as in the case of DAPI, of some weak, nonspecific attraction. However, when the target genomic DNA of *E. coli* bl21-de3 was introduced, only the MMV-PS particles formed large aggregates with strong fluorescence (Fig. 3*A2*), revealing its high affinity toward the target analyte. In contrast, the PolyA-PS and RAN-PS colloids both produced only tiny aggregates (Fig. 3*B2* and *C2*) with a size that was some 50 times smaller than those formed by MMV-PS colloids, as can be seen from the graphs in (Fig. 3*D*).

### Sensitivity and Specificity of the Biosensor.

The sensitivity of a pathogen detection method is important because early identification of the pathogen, at a time when the levels are still low, is crucial for informing the subsequent treatment strategy. To test the sensitivity of our multivalent colloidal biosensor to the presence of the target genome, we monitored how the size and fluorescence intensity of the aggregate varied with the addition of increasing amounts of target genome, ranging from $10^{1}$ to $10^{10}$ copies/mL. A sample containing only MMV-PS and DAPI with no genomic DNA was used as a blank control.

**Fig. 4.**
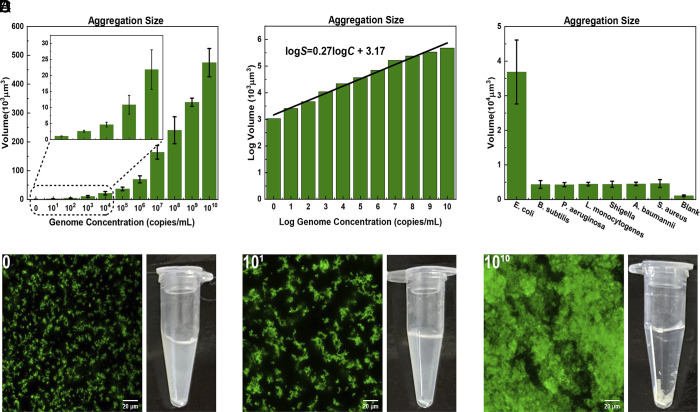
Detection limits of the proposed biosensor. (*A*) Histogram showing the variation of average aggregate sizes as function of genome concentration (ranging from 10 copies per mL to $10^{10}$ copied per mL. The *Left-most* points in plots (*A*) and (*B*) correspond to genome concentration of 0, rather than 10${}^{0}$. The solid bars depict the average size of the aggregate. In this graph, we indicate the estimated SD based on three independent measurements. (*B*) Fitted calibration curve for the average aggregation sizes of the logarithmically plotted data in (*A*). (*C*) The specificity of the proposed biosensor to the genome of *E. coli* and other bacterial genomes. The genome concentration in each sample was $10^{5}$ copies/mL. (*D*) Confocal microscope images and corresponding Eppendorf tubes of a pure MMV-DNA/DAPI sample (control sample), one containing 10 and one with $10^{10}$ genome copies per ml.

As shown in Fig. 4*A*, the aggregate size grows with the concentration of the target genome from $∼10^{4}$μm${}^{3}$ at a target concentration of $10^{3}$ copies/mL, to $∼5\times 10^{5}$ μm${}^{3}$ at $10^{10}$ copies/mL. Even for genome concentrations as low as 10 copies/mL, the observed fluorescent patches were already significantly larger than for the blank control (Fig. 4*D*).

The log-log plot in Fig. 4*B* suggests that over a rather wide concentration range, the relation between aggregate size S and genome concentration C is well approximated by a power law of the form $∼C^{0.27}$. In the log–log plot: logS≈0.27logC+3.17 with R${}^{2}$ = 0.985. In the above equation, S is expressed in μm${}^{3}$, and C is measured in units of copies/mL. On the basis of this figure, we estimate the limit of detection (LoD) to be around 5 genome copies/mL, which is the value of the genome concentration where the extrapolated log–log relation crosses the cluster volume of the blank sample (27, 28). However, this LoD applies to the case where nontarget genome is absent. A more relevant measure of the LoD is the ability to distinguish the aggregate sizes formed by target and nontarget genomes. As can be seen from Fig. 4*C*, 10${}^{5}$ copies/mL of nontarget genome induce the same size of aggregates as 10${}^{2}$ copies/mL of the target genome. For samples with a background bacterial concentration less than 10${}^{5}$ copies/mL, the de facto limit of detection of target genome is therefore around 10${}^{2}$ copies/mL. Importantly, as viral genomes are typically about two orders of magnitude shorter than bacterial genomes, superselective binding of viral DNA is expected to be much weaker than for bacterial DNA.

Overall, the LoD of our full-genome approach compares favorably with some of the detection methods currently in use (Table 1). These results suggest that the multivalent-colloid detection system has the capability to detect pathogens at an early stage when the bacterial load is still low. Table 1 summarizes the performance of our proposed genome detection strategy compared with other detection methods (29–33).

**Table 1.** Comparison of different *E. coli* detection methods with fluorescent signal output in recent years

Target	Method	Limit of detection	Linear range	Reaction time
Extracellular proteins (29)	DNAzyme	$10^{3}$ CFU/μL	–	Mix and read
Cell/lipopolysaccharides (30)	Aptamer	487 CFU/mL	500-$10^{6}$ CFU/mL	40 min
Cell/mycoproteins (31)	Polyclonal antibody	$10^{3}$ CFU/mL	$10^{3}$–$10^{5}$ CFU/mL	25 min
Target gene (32)	PCR	10 copies	10-$10^{5}$ copies	100 min
Target gene (33)	RPA	100 copies	$10^{2}$–$10^{7}$ copies	13 min
Whole genome (present work)	Designed primers	∼5 copies/mL	10-$10^{10}$ copies/mL	Mix and read

Here, PCR, RPA for recombinase polymerase amplification, and CFU for colony-forming units. The number of CFU is typically less than the number of microorganisms deposited on a plate containing growth medium.

### Selectivity.

In order to detect a genome reliably, we require not only sensitivity but also selectivity: Nontarget genomes should induce a response above the background. In this context, we found once again that the crucial discriminator between target and nontarget genome was not so much the presence of colloidal clusters, but the size of these clusters (*SI Appendix*, Fig. S9).

We tested the selectivity of the multivalent colloid detection system by measuring its response to a number of nontarget genomes using the experimental conditions developed for the *E. coli* bl21-de3 genome. The pre-extracted genomic DNAs of 6 counter targets (3 gram-positive and 3 gram-negative) were denatured using the method described in the *Experimental*. We considered the situation where the number of bacteria in the sample might be small, even after centrifugation. Therefore, we mixed samples with different types of genomic DNA at a concentration of $10^{5}$ copies/mL, with a fixed amount of MMV-PS particles. As can be seen from Fig. 4*C* (and *SI Appendix*, Fig. S9), the genomic DNA of *E. coli* bl21-de3 was found to lead to much larger particle aggregates (almost 10 times larger) than the clusters that formed in solutions of the six nontarget genomes at the same concentration. The fact that nontarget DNA-induced cluster formation indicates that the MMV-PS colloids could attach to nontarget genomes, but much less than to the target genome. To quantify the difference, we found that the clusters obtained in solutions of $10^{5}$ copies/mL nontarget genome were comparable in size to those found at a one thousand times lower concentration of the target genome. These observations illustrate that the current detection strategy can achieve very good, though not perfect selectivity.

### Testing the MMV Probes in a Human Urine Sample.

**Fig. 5.**
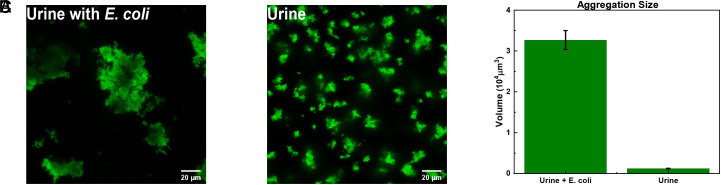
Testing our optimized multivalent colloidal probes plus DAPI fluorophore sensor in human urine samples. (*A*) Human urine sample containing $10^{5}$ copies/mL of the target genome *E. coli* bl21-de3. (*B*) The fresh urine sample shows many tiny aggregations. (*C*) Comparison of aggregation sizes between samples with and without the target genome.

In order to verify the analytical reliability and practical potential of the biosensor for pathogen detection in clinical samples, human urine from a healthy individual spiked with trace amounts of *E. coli* bl21-de3 was selected as a model sample. Specifically, two samples were prepared: One was pure urine (*Experimental*), whereas the other contained the target bacteria at a concentration of $10^{5}$ copies/mL. Both samples contained concentrations of the MMV probes and DAPI similar to the ones used in our proof-of-principle experiments. The genome extraction procedure was performed on both samples. As can be seen from Fig. 5 *A* and *B*, the sample spiked with the target genome showed much larger clusters than those found in the pure urine sample.

Analyzing confocal image stacks of the pure urine sample containing the optimized mix of MMV-probes and DAPI molecules showed small clusters that were similar in size to those observed in our control samples (Fig. 4*D*). The cluster sizes in the spiked urine sample, on the other hand, were similar to those found for the samples containing $10^{5}$ copies/mL of genomic DNA in Fig. 4*C*. This result suggests that the calibration curve for our optimized MMV probe plus DAPI system, established in buffer solution (Fig. 4*B*), can act as a guideline to real samples.

## 2. Conclusions

In this paper, we have tested the genome detection strategy that had been proposed on the basis of calculations by Curk et al. (7). We find that immobilization of the surface DNA probes onto PS colloids results in particle aggregation with strong fluorescence upon the introduction of genomic DNA of the target bacteria. This fluorescence can be readily observed without prior PCR amplification. The DNA-mediated biosensor has several attractive features that distinguish it from existing biosensors. First of all, once the analyte is injected to react with DNA-functionalized PS particles, it requires less than 1 min to obtain the results, which is significantly faster than existing strategies with comparable sensitivity. Moreover, our system is able to detect non-PCR-amplified genomes over a genome concentration range spanning 10 orders of magnitude. We estimate a lower limit of detection in the absence of other bacterial genomes at ∼5 genome copies/mL.

The proposed biosensor performed well in distinguishing target and nontarget bacteria from both pure cultures and from spiked human samples. The biosensing technique based on whole-genome computational algorithms can be applied to detect a wide range of pathogens (bacteria and fungus) by simply grafting PS colloids with corresponding matching surface DNA probes. The limitation is that the approach will not work for short genomes, which is common for viruses. However, that disadvantage is an advantage because it would imply that viral infections can be readily distinguished from bacterial infections.

We stress that the present paper describes only a first set of tests. Clearly, there is still much space to optimize our biosensing technique. We hope that the results that we have presented show that in view of the many possible applications, in particular in low-tech environments, it would be highly desirable to further develop the multivalent colloidal detection system for practical applications.

## Supplementary Material

Appendix 01 (PDF)

## Data Availability

Digitized confocal microscopy images data have been deposited in Zenodo (10.5281/zenodo.8127777). All study data are included in the article and/or *SI Appendix*.
