# Supplementary material for: Whole-genome detection using multivalent DNA-coated colloids
Source: Proc Natl Acad Sci U S A. 2023 Sep 5;120(37):e2305995120. doi: 10.1073/pnas.2305995120 (PMC10500262; doi:10.1073/pnas.2305995120)
Supplement: Supplementary file 1 — Appendix 01 (PDF) [file pnas.2305995120.sapp.pdf]

Supporting Information for

Whole Genome Detection using Multivalent DNA-Coated Colloids

Peicheng Xu, Ting Cao, Qihui Fan, Xiaochen Wang, Fangfu Ye, Erika Eiser

Erika Eiser and Fangfu Ye.
E-mail: Erika.Eiser@ntnu.no, fye@iphy.ac.cn

This PDF file includes:

Supporting text
Figs. S1 to S9
SI References

Supporting Information Text

Optimization of the biosensor

Several factors influence the performance of the MMV-PS probe colloids. We tested the effect of varying the concentration of the PS-b-PEO-N_3 diblock-copolymer, which anchors the MMV-probe strands to the particle surface (see subsection (A) below), the effect of varying grafting densities of the ssDNA probes (B), the effect of changing the Cu^{2+} concentration in the solution (C), and the effect of changing the salt (NaCl) concentration (D).

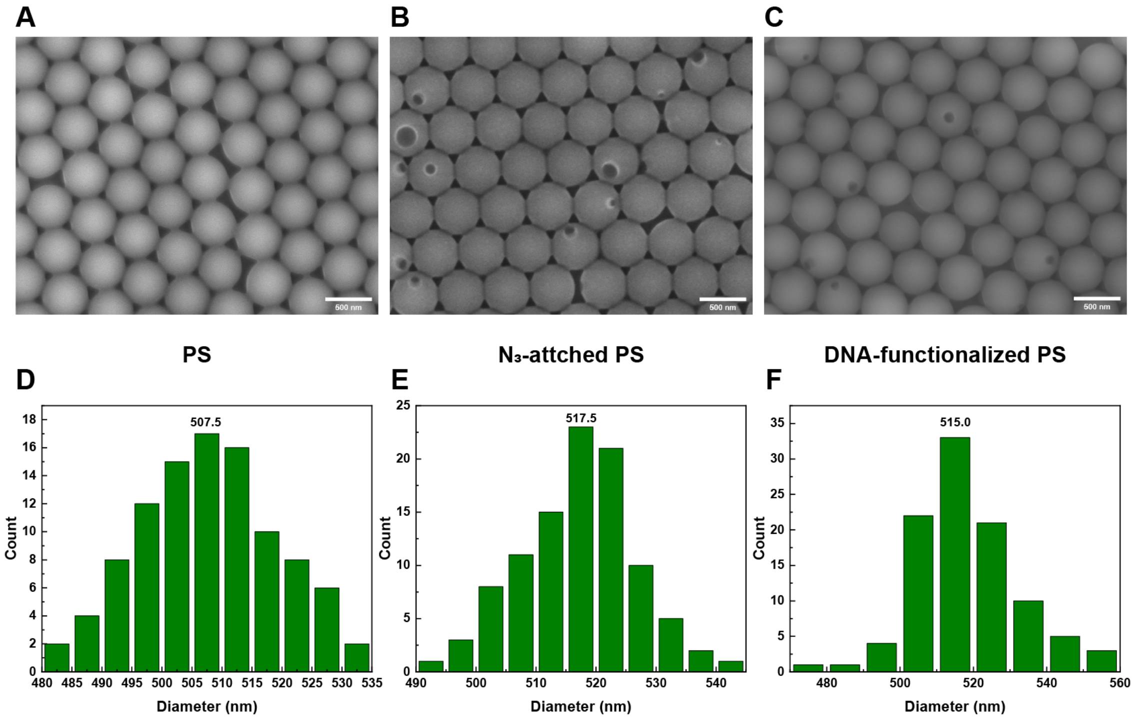

Fig. S1. SEM images of (A) plain, surface-charged PS particles, (B) those functionalized with PS-b-PEO-N_3 and (C) after attachment of the 20 nucleotide-long ssDNA to the PEO-N_3 ends via click chemistry. (D)-(F) Corresponding size distributions of the bare and functionalized PS particles. The scale bars correspond to 500 nm.

A. Grafting density of the PS-b-PEO-N_3 on the PS-colloid surfaces. The grafting density of the MMV-DNA strands per colloid is controlled by the grafting density of the PS-b-PEO-N_3 (abbreviated as PS-PEO-N_3) diblock copolymer on the colloid surfaces. We used a protocol by Oh et al. (1), modified and characterized by the Eiser group (2–4), in which the PS particles are swollen with THF such that the short, hydrophobic PS-blocks can penetrate into the particles, while the water-soluble PEO-N_3 blocks remain solvated in the aqueous phase. By subsequent replacement of the aqueous THF-solution with deionized water the swollen PS particles deswell and return into their glassy state, thereby locking the PS-blocks in. Varying the block-copolymer solution from zero to 2 mM, we find that at the optimal block-copolymer to colloid concentration ratio (here it is $1{:}10^8$) we have a grafting density of 12 nm^2 per chain, which corresponds to $\sim 7 \times 10^4$ chains per 513 nm large PS-bead (4). At such a grafting density the block-copolymer forms a sterically stabilizing polymer brush of only a few nanometer thickness (Fig. S1), which suppresses non-specific colloid-colloid interactions such as van der Waals (5) or Coulomb. In all following tests and the results presented in the main article we used this highest block-copolymer grafting density. Note that attaching the ssDNA strands to the PEO-brush layer did not seem to further increases the overall diameter of the colloids (Fig. S1F).

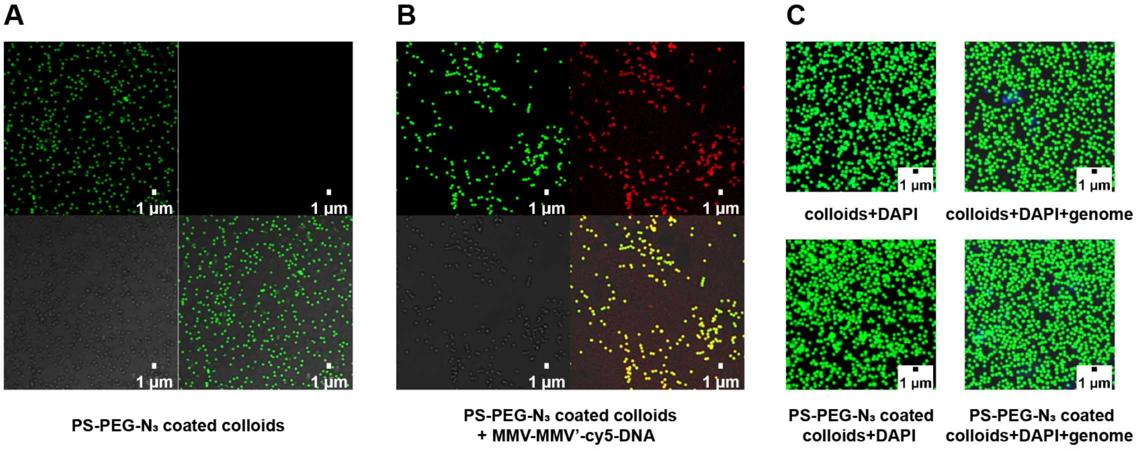

Fig. S2. Confocal images of (A) our green-fluorescent PS particles, coated with a dense brush of PS-b-PEO-N_3 diblock copolymers recorded in the green (top left), red (top right), bright field (bottom left) and superposed (bottom right) imaging mode. (B) Confocal microscopy images of the same type particles after functionalizing them with ssDNA and subsequently hybridizing them with complementary, red-fluorescent, cy5-ssDNA. The same imaging modes were used as in (A). (C) The effect of DAPI staining: Confocal microscope images of DAPI stained plain PS-particle solutions without (top left) and with the target genome (top right), and polymer-brush coated particle solutions without (bottom left) and with the target genome (bottom left). Here we show the overlay of the green and blue fluorescent channel.

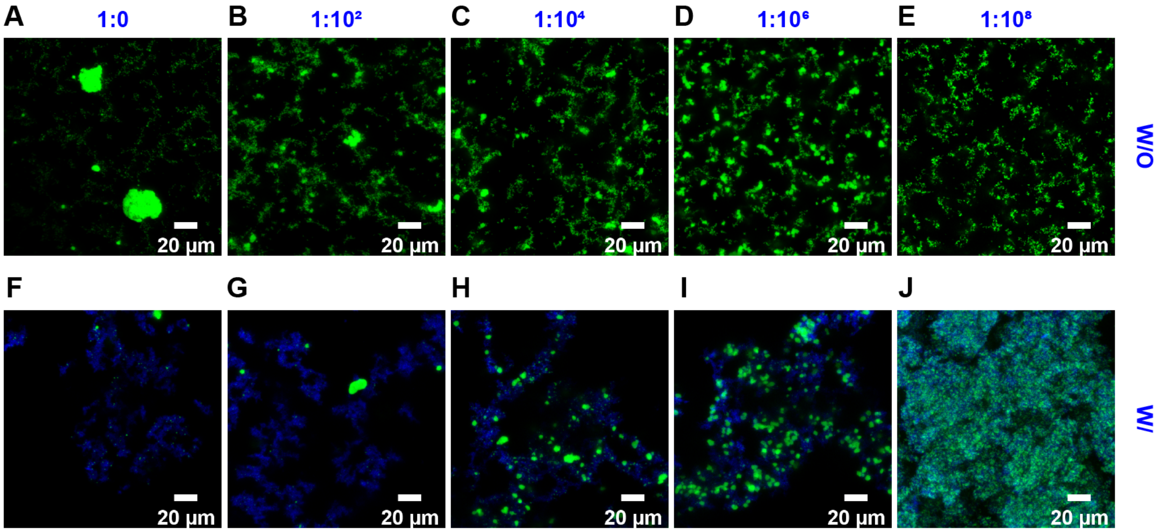

Fig. S3. Effect of PS-b-PEO-N_3 grafting density on the colloidal aggregation in the absence (W/O) and presence (W/) of the *E.coli* bl21-de3 genome. Confocal microscopy images of solutions of bare colloids (A), and those coated with a grafted block-copolymers using a preparation of 1:10^2 colloids per PS-b-PEO-N_3 chains up to 1:10^8 colloids per PS-b-PEO-N_3 chains (B-E) measured in the presence of the target genome, and without (F-J) the target genome. Here, E8 was prepared by mixing 2000 µL PS-PEO-azide (2.5mM) with 150 µL PS (10g/L). All samples contained 1g/L DAPI.

B. Covalent bonding of ssDNA probes to the PS-b-PEO-N_3 brush. The covalent attachment of the alkyne-termiated ssDNA strands was performed using the Copper(I) catalysed azide(N_3)-alkyne cycloaddition, which is a click reaction that works in water; the reaction is fast, highly efficient over a range of pH's and temperatures, produces minimal by-products and has yields >90% (4, 6). The efficacy of this MMV-DNA attachment was tested by hybridizing them with complementary ssDNA strands carrying a red fluorescent cy5-MMV´-DNA. In Fig. S2 we show confocal images of the green fluorescent PS-particles, functionalized with the block-copolymer brush and after attachment of the probe DNA, reacted with the red-fluorescent complimentary MMV´-DNA.

The propensity of our bare, green PS-colloids and those grafted with different PS-PEO-N_3-brush densities were tested for self-aggregation, both in the absence (W/O) and presence (W/) of the target genome. Note that all solutions were prepared in PBS buffer containing ascorbic acid and 1g/L of the DAPI fluorophore. The results are shown in Fig. S3. A comparison between the confocal images Fig. S3A and F, of suspension prepared in the solvent conditions used in the final, optimal probe solutions, it is clear that DAPI promotes strong aggregation of the bare PS-colloids in the absence of the genome. This colloidal aggregation is almost completely suppressed in the presence of the genome, which is due to the fact that the DAPI preferentially interacts with the genomic DNA, even after it has been denatured. This is due to the fact that locally hybridization between the long DNA sequences can occur. Note that in Fig. S3 we only show the green fluorescent channel in the confocal images. Monitoring also the blue DAPI fluorescence, indeed indicates weak but negligible DAPI-DNA binding.

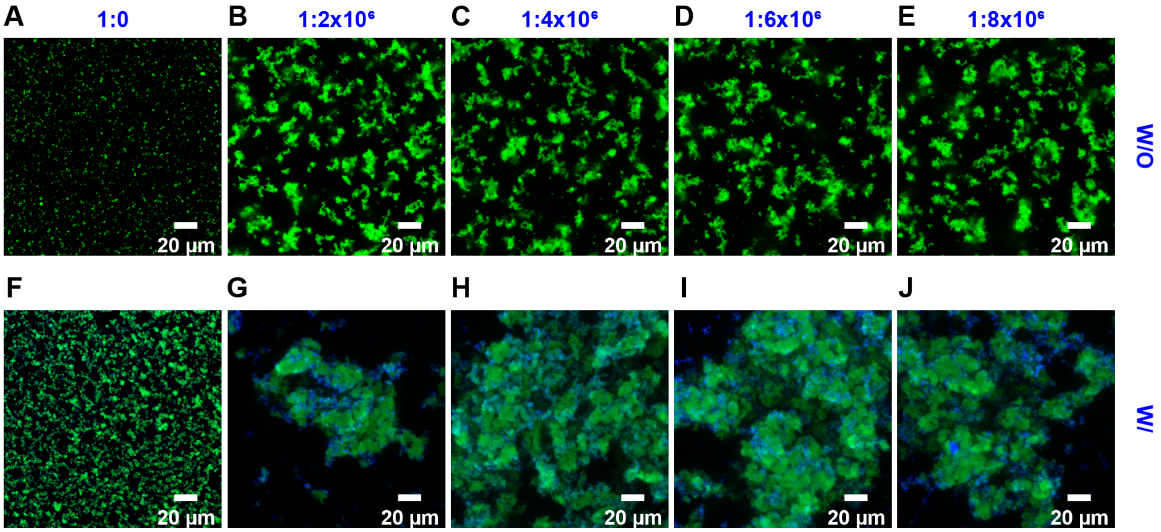

Fig. S4. Testing the MMV-DNA grafting density on the probe colloids. Overlay of green and blue fluoresence confocal microscope images taken in the absence of the denatured *E. coli* genome for (A) colloids grafted only with the PS-PEO-N_3 brush and (B-E) for increasing colloid to MMV-DNA concentrations after the click-reaction and removal of excess DNA and reaction products. All images were taken from solutions containing DAPI. In the presence of genome the confocal images show (F-J) that with increasing MMV-grafting density on the colloid surface the effective cluster size is increasing while keeping the genome density fixed.

In Fig. S4 we show how the MMV-DNA grafting density on the colloid-PEO-N_3 brush influences the binding of the probes with the target bacterial genome. The alkyne-modified ssDNA probes are attached to the colloidal surface using a click reaction with the azide group of the PS-b-PEO-N_3 block copolymers on the colloidal surface. The concentration of the alkyne-modified ssDNA probes in solution determines the resulting surface coverage of ssDNA probes reacted to the PEO-azide chain ends. In our tests, the number of grafted DNA probes per colloid was varied by varying the ratio of colloids per MMV-DNA concentration from 1:0 to 1:8 x 10^6. In Fig. S4 we see that we see some aggregarion of the DNA-coated colloids in the presence of 1 g/L DAPI, which we found to promote weak aggregation between the ssDNA strands on the colloids. However, this aggregation is negligible compared to the cluster growth in the presence of the target genome, as is visible in Fig. S4F-J: The aggregation size increases with the surface coverage of the DNA probes, and saturates when the number of DNA probes per colloid exceeds $\approx$4 x 10^6. This coverage was achieved by exposing the azide-functionalized colloids to a DNA probe solution with a concentration of 5 μM. We note that the pure PS-PEO-N_3 coated colloids formed very few, negligibly small aggregates, even in the presence of DAPI (Fig. S4A). In view of the outcome of the above tests, we fixed the DNA surface coverage of the PS colloids at 4 x 10^6 in all subsequent experiments.

C. Effect of Cu^{2+}, necessary for the click-reaction. Another parameter that affects the performance of the multivalent genome detection is the Cu^{2+} concentration used in the click reaction to graft the ssDNA probes onto the azide ends of the PEO brush on the colloids. We carried out the click reaction using Cu^{2+} concentrations varying from 0 to 20000 μM, while the corresponding concentration of ascorbic acid was five times higher. As can be seen from Fig. S5, an increase of the Cu^{2+} concentration during the preparation of the MMV-DNA grafted colloids resulted in an increase of the aggregate size and fluorescence intensity of the resulting DNA-coated colloids, when these were mixed with target genome. However, the aggregate size reached a peak when the click reaction was carried out at a Cu^{2+} concentration of 2 mM. Higher Cu^{2+} concentrations appeared to damage ssDNA, resulting in a decreased binding between the colloids and the target genome (Fig. S6A). DNA damage due to the presence of Cu^{2+} ions has been reported earlier (7). When the click reaction was carried out at much lower Cu^{2+} concentrations, only few DNA probes could be grafted onto PS particles and the binding of the colloids to the targeted genome was strongly suppressed (Fig. S5B for the 20 mM Cu^{2+} concentration).

Based on the above findings, the Cu^{2+} concentration of 2 mM used for the click reaction did not seem to affect the aggregation behaviour of colloids in the presence of the target genome, and thus was used for all further experiments.

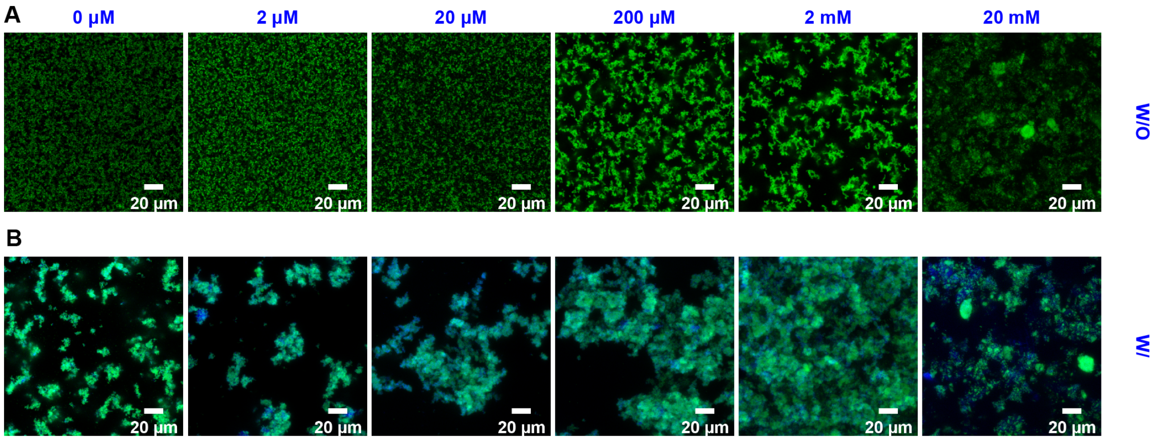

Fig. S5. Effect of the Cu^{2+} concentration on the click-reaction. Like in Fig. S4 we show the overlay of green and blue fluoresence confocal microscope images taken in the absence of the denatured *E. coli* genome for (A) colloids grafted with the PS-PEO-MMV brush for increasing Cu^{2+} concentration used in the click-reaction to attach MMV-DNA (4 x 10^6) to the azide ends of the PEO brush. All images were taken from solutions containing 1 g/L DAPI. (B) In the presence of the genome the confocal images show that the largest clusters are observed using a 2mM Cu^{2+} concentration for the click reaction.

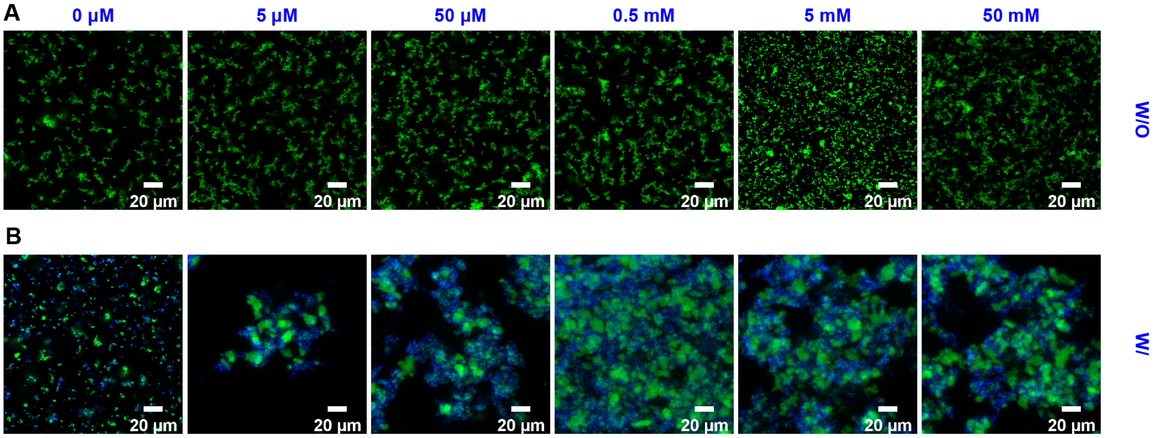

Fig. S6. Effect of the added NaCL concentration on the hybridization efficiency of the MMV-probes with the target genome. Like in Fig. S5 we show the overlay of green and blue fluorescence confocal microscope images taken in the absence of the denatured *E. coli* genome for (A) colloids grafted with the PS-PEO-MMV brush for increasing added NaCl in the presence of 2 mM Cu^{2+} and best MMV coverage found in Fig. S4. All images were taken from solutions containing 1 g/L DAPI. (B) In the presence of the genome the confocal images show that the largest clusters are observed using 0.5 mM added NaCl.

D. Effect of added NaCl. The 1:1 salt NaCl is typically needed to screen the electrostatic repulsion between the negatively charged sugar-phosphate backbones of the DNA single strands, in order for them to be able to bind to each other via hydrogen bonds. We explored what range of NaCl concentrations is needed for optimal genome detection.

In Fig. S6B we can see that in the presence of the target genome, the size of colloidal aggregates initially increases with salt concentration and then slightly decreases as the salt concentration is varied from 0 to 50 mM. In the absence of NaCl, small aggregates still formed since DAPI is also a salt. Furthermore, the present Cu^{2+} concentration adds to the overall screening of the negative charge along the ssDNA backbones. Note that the electrostatic repulsion between equal charges is stronger for divalent than of monovalent salts such as NaCl (8). The size of aggregates reached a maximum value for a NaCl concentration of ≈ 0.5 mM, and decreased slightly beyond that concentration. Hybridization (binding) and dehybridization (unbinding) measurements of short, complementary ssDNA sequences as function of added NaCl concentration showed that only for concentrations ≥ 50 mM in deionized water equilibrium hybridization was observed (9). Below that concentration a hysteresis in the melting/heating curves is observed. As we have also Cu^{2+} and DAPI are present the overall ionic strength of the final solution containing the probe colloids and genome is still a bit lower that that 50 mM limit. The melt temperature of a complementary duplex decreases slightly with increasing salt. Therefore, we can assume that in our case the final ionic strength should be a bit lower than that for equilibrium hybridization, meaning the weaker screening of the negative backbone charges helps the MMV-probes to bind and unbind with greater probability than for higher ionic strengths, enabling the system to reach its lowest binding-free energy. In other words, upon further increasing the NaCl concentrations leads to stronger binding energies and thus kinetically slowing down the formation of the maximum number of base-pairs due to kinetic hin. This kinetic slowing down may lead to the smaller clusters we observe at higher NaCl concentrations. Note that all samples are mixed at room temperature. The influence on aggregation was also studied earlier on suspensions of DNA-coated fd-viruses binding to each other via gold-nanoparticles, coated with the complementary ssDNA (10).

Further, we find that the added NaCl concentration has little effect on the size of the colloidal clusters formed in the absence of genome Fig. S6A. Thus, in all results shown in the main text we used the optimum salt concentration of 0.5 mM NaCl. In this work the aggregation type was discussed as function of quenching rates below the systems melting temperature.

Choice of control parameters

In Fig. S7 we present bar charts of the size of the aggregated colloidal clusters formed, when testing the four different parameters presented above. While we show the overlayed confocal images of the green and blue fluorescence of the colloids and the DAPI interacting with the genomic DNA in Figs. SI3-SI6, we extracted the observed cluster sizes using only the green fluorescence of the colloids, avoiding exciting the DAPI fluorophore in order to minimize possible cross-talk. The aggregate sizes were extracted from stacks of confocal images, each representing a thickness $\Delta z = 1.48$ μm, as described in the main text.

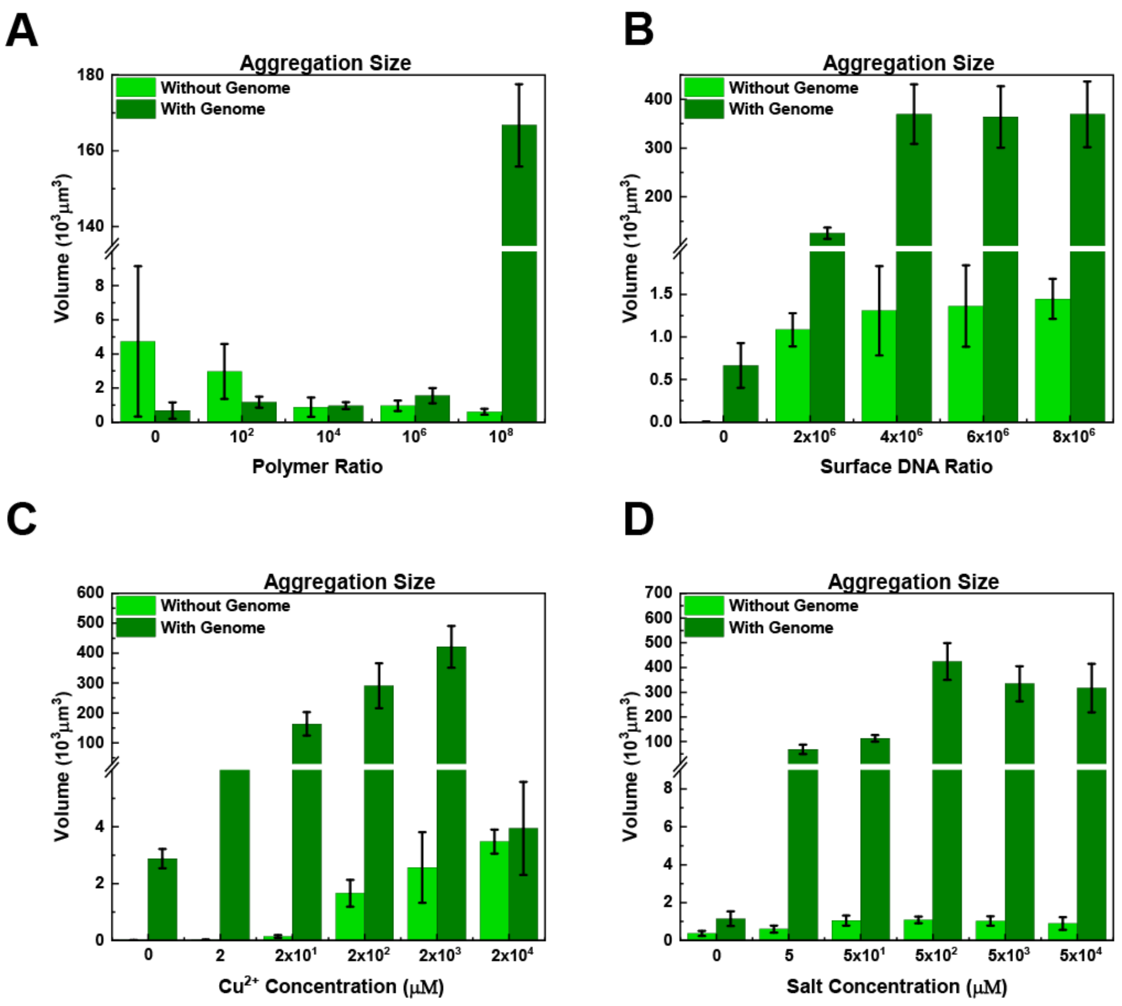

Fig. S7. Effect of four control parameters on the performance of the proposed biosensor. The operating conditions used in the main text were selected on the basis of the above data. The relation between the observed aggregate size of PS colloids as a function of (A) polymer ratio, (B) DNA probe ratio, (C) Cu^{2+} concentration, (D) salt concentration. Note that, in order to represent genomic and non-genomic data on the same plots, the figures have a scale change of more than two orders of magnitude

101 ## Stability of the MMV-colloidal probes

In Fig. S8 we show confocal images in which we tested the stability of our MMV-probes against degradation in time.

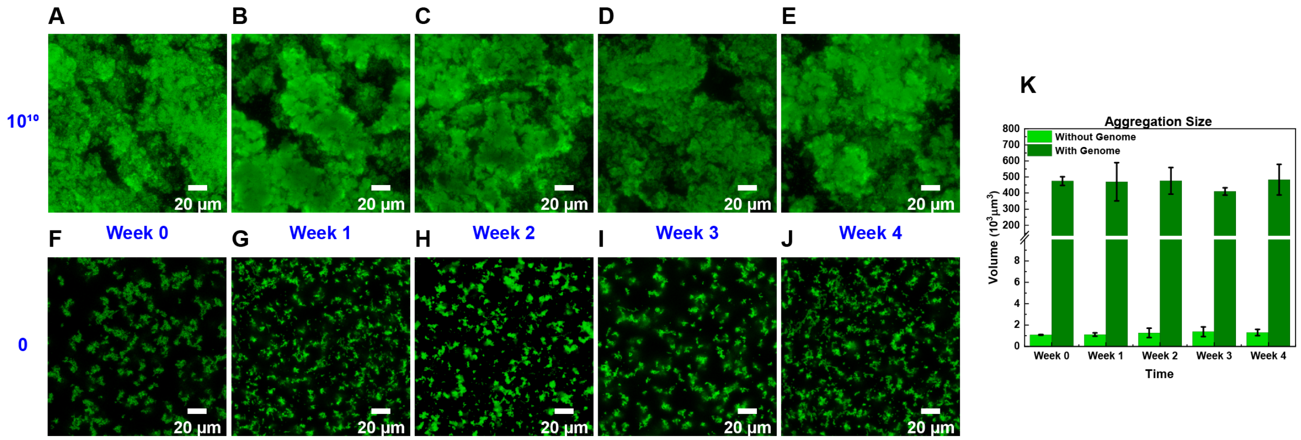

Fig. S8. Stability of the proposed biosensor. Confocal microscopy images, showing green fluorescence of the probe-colloids only, of the formation of genome-induced aggregates for different times after the preparation of the DNA-functionalized probes. Frames (A-E) show the aggregates formed upon addition of the target genome after 0 to 4 weeks. Here we used a genome density of 10^{10} copies/mL. Frames (F-J) illustrate the absence of large-scale aggregation of the pure MMV-coated colloidal probe solutions over the same period. All samples here contained a 1 g/L DAPI concentration.

102

Selectivity of the MMV-colloidal probes

We tested the selectivity of our MMV-probes, designed to recognize the *E.coli* bl21-de3 genome, by mixing these probes with the genomes of six other bacteria. Using the solvent condition chosen for detecting our *E.coli* strain, we observe that even for hundred-fold higher concentrations of the other bacterial genomes we do not observe comparably strong colloid aggregation (Fig. S9).

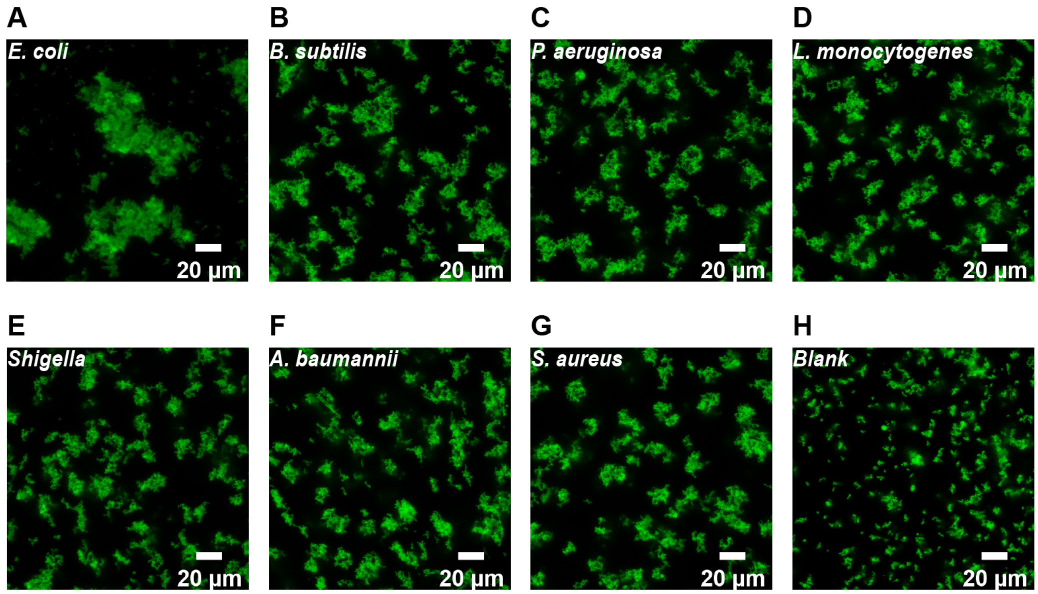

Fig. S9. Confocal images, comparing the selectivity of our MMV-probes for the *E.coli* bl21-de3 genome with six other bacterial genomes. Here we used an *E.coli* concentration of 10^5 copies/mL, while all other samples contained 10^7 copies/mL.

References

1. JS Oh, Y Wang, DJ Pine, GR Yi, High-density peo-b-dna brushes on polymer particles for colloidal superstructures. *Chem. Mater.* **27**, 8337–8344 (2015).
2. M Zupkauskas, Y Lan, D Joshi, Z Ruff, E Eiser, Optically transparent dense colloidal gels. *Chem. Sci.* **8**, 5559–5566 (2017).
3. A Caciagli, et al., Dna-coated functional oil droplets. *Langmuir* **34**, 10073–10080 (2018).
4. T O'Neill, Ph.D. thesis (University of Cambridge, Cavendish Laboratory, J. J. Thomson Avenue, Cambridge CB3 0HE, U.K.) (2019).
5. SC Tsai, K Zammouri, Role of interparticular van der waals force in rheology of concentrated suspensions. *J. Rheol.* **32**, 737–750 (1988).
6. C Spiteri, JE Moses, Copper-catalyzed azide–alkyne cycloaddition: regioselective synthesis of 1, 4, 5-trisubstituted 1, 2, 3-triazoles. *Angewandte Chemie Int. Ed.* **49**, 31–33 (2010).
7. L Cai, J Koropatnick, MG Cherian, Roles of vitamin c in radiation-induced dna damage in presence and absence of copper. *Chem. Interactions* **137**, 75–88 (2001).
8. JN Israelachvili, *Intermolecular and surface forces.* (Academic press), (2011).
9. Z Xing, et al., Microrheology of dna hydrogels. *Proc. Natl. Acad. Sci.* **115**, 8137–8142 (2018).
10. Z Ruff, et al., Designing disordered materials using dna-coated colloids of bacteriophage fd and gold. *Faraday discussions* **186**, 473–488 (2016).
